# 2-(4-Chloro-*N*-{2-[(1*H*-pyrrol-2-yl)carbon­yloxy]eth­yl}anilino)ethyl 1*H*-pyrrole-2-carboxyl­ate

**DOI:** 10.1107/S1600536811056170

**Published:** 2012-01-14

**Authors:** Ying Yan, Guilong Zhang, Zhenming Yin

**Affiliations:** a Tianjin Key Laboratory of Structure and Performance for Functional Molecules, College of Chemistry, Tianjin Normal Uinversity, Tianjin 300387, People’s Republic of China; b Agro-Environmental Protection Institute, Ministry of Agriculture, Tianjin 300191, People’s Republic of China

## Abstract

In the title mol­ecule, C_20_H_20_ClN_3_O_4_, both the pyrrole N—H groups adopt a *syn* conformation with respect to the carbonyl groups. In the crystal, inter­molecular N—H⋯O hydrogen bonds link the mol­ecules into layers parallel to (102).

## Related literature

For the crystal structures of related pyrrole-2-carboxyl­ate derivatives, see: Sessler *et al.* (2003 ▶); Yin & Li (2006 ▶); Maeda *et al.* (2007 ▶); Cui *et al.* (2009 ▶).
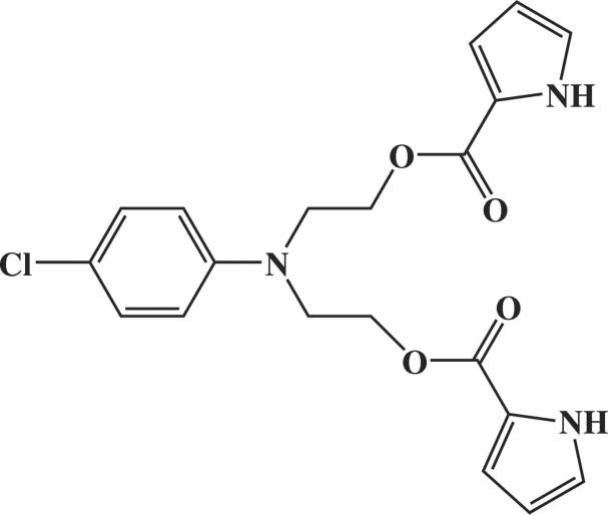


## Experimental

### 

#### Crystal data

C_20_H_20_ClN_3_O_4_
*M* *_r_* = 401.84
Monoclinic, 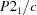

*a* = 19.972 (2) Å
*b* = 4.7426 (5) Å
*c* = 20.613 (2) Å
β = 95.815 (2)°
*V* = 1942.4 (4) Å^3^
*Z* = 4
Mo *K*α radiation
μ = 0.23 mm^−1^
*T* = 296 K
0.28 × 0.20 × 0.18 mm

#### Data collection

Bruker SMART CCD area-detector diffractometer
Absorption correction: multi-scan (*SADABS*; Bruker, 1999 ▶) *T*_min_ = 0.488, *T*_max_ = 1.000
9198 measured reflections
3438 independent reflections
1763 reflections with *I* > 2σ(*I*)
*R*_int_ = 0.046

#### Refinement

*R*[*F* ^2^ > 2σ(*F* ^2^)] = 0.038
*wR*(*F* ^2^) = 0.106
*S* = 1.01
3438 reflections
254 parameters
H-atom parameters constrained
Δρ_max_ = 0.15 e Å^−3^
Δρ_min_ = −0.24 e Å^−3^

### 

Data collection: *SMART* (Bruker, 1999 ▶); cell refinement: *SAINT* (Bruker, 1999 ▶); data reduction: *SAINT*; program(s) used to solve structure: *SHELXS97* (Sheldrick, 2008 ▶); program(s) used to refine structure: *SHELXL97* (Sheldrick, 2008 ▶); molecular graphics: *SHELXTL* (Sheldrick, 2008 ▶); software used to prepare material for publication: *SHELXTL*.

## Supplementary Material

Crystal structure: contains datablock(s) global, I. DOI: 10.1107/S1600536811056170/cv5220sup1.cif

Structure factors: contains datablock(s) I. DOI: 10.1107/S1600536811056170/cv5220Isup2.hkl

Supplementary material file. DOI: 10.1107/S1600536811056170/cv5220Isup3.cml

Additional supplementary materials:  crystallographic information; 3D view; checkCIF report

## Figures and Tables

**Table 1:** Hydrogen-bond geometry (Å, °)

*D*—H⋯*A*	*D*—H	H⋯*A*	*D*⋯*A*	*D*—H⋯*A*
N1—H1⋯O1^i^	0.86	2.07	2.893 (3)	160
N3—H3⋯O4^ii^	0.86	2.07	2.891 (3)	158

Symmetry codes: (i) 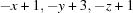; (ii) 
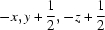
.

**Fig. 1.**
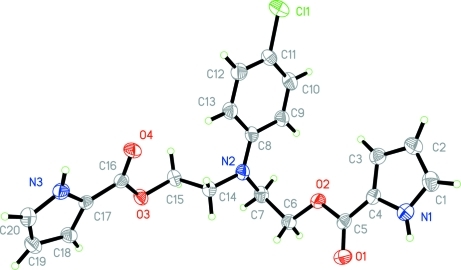
The molecular structure of (I), with the atom-numbering scheme and 30% probability displacement ellipsoids.

## supplementary crystallographic information

### Comment

The use of 2-carbonyl-functionalized pyrrole moieties as building blocks to create hydrogen bonded self-assembled aggregates has received some attention recently (Sessler *et al.*, 2003; Yin *et al.* 2006; Maeda *et al.* 2007). In continuation of our study of the solid state self-assemblies of some pyrrole-2-carboxylate compounds (Cui *et al.* 2009), we report the crystal structure of the title compound, (I).

In (I) (Fig. 1), both the pyrrole NH groups adopt *syn* conformation with respect to the carbonyl groups. The molecules of the title compound self-assemble into one-dimensional tape through helical N—H···O hydrogen bonds (Table 1). Further, intermolecular N—H···O hydrogen bonds (Table 1) link these tapes into layers parallel to (102) plane. The hydrogen bonding motif in the crystal of (I) is different from that reported for N,*N*-di[2- (1*H*-pyrrole-2-carbonyloxy)ethyl]-aniline (Cui *et al.*, 2009). Apparently, the chloro group has great influence on the crystal packing.

### Experimental

*N*,*N*-Di(2-hydroxyethyl)-4-chloroaniline (0.215 g), 2-(trichloroacetyl)-1*H*-pyrrole(0.59 g) and triethylamine (1 mL) were added to acetonitrile (15 ml), and the mixture was refluxed for 10 h. The solution was then evaporated under reduced pressure and the residue was purified by column chromatography on silica gel with ethyl acetate-petroleum ether (1:4 *v*/*v*), affording the title compound.

### Refinement

All H atoms were geometrically positioned (N—H 0.86 Å, C—H 0.93-0.97 Å), and included in the final cycles of refinement using a riding model, with *U*_iso_(H) = 1.2 *U*_eq_(C, N).

### Crystal data

**Table:**

C_20_H_20_ClN_3_O_4_	*D*_x_ = 1.374 Mg m^−^^3^
*M**_r_* = 401.84	Melting point: 438 K
Monoclinic, *P*2_1_/*c*	Mo *K*α radiation, λ = 0.71073 Å
*a* = 19.972 (2) Å	Cell parameters from 1561 reflections
*b* = 4.7426 (5) Å	θ = 2.7–21.7°
*c* = 20.613 (2) Å	µ = 0.23 mm^−^^1^
β = 95.815 (2)°	*T* = 296 K
*V* = 1942.4 (4) Å^3^	Block, yellow
*Z* = 4	0.28 × 0.20 × 0.18 mm
*F*(000) = 840	

### Data collection

**Table:**

Bruker SMART CCD area-detector diffractometer	3438 independent reflections
Radiation source: fine-focus sealed tube	1763 reflections with *I* > 2σ(*I*)
graphite	*R*_int_ = 0.046
phi and ω scans	θ_max_ = 25.0°, θ_min_ = 1.0°
Absorption correction: multi-scan (*SADABS*; Bruker, 1999)	*h* = −23→23
*T*_min_ = 0.488, *T*_max_ = 1.000	*k* = −5→5
9198 measured reflections	*l* = −24→13

### Refinement

**Table:**

Refinement on *F*^2^	Secondary atom site location: difference Fourier map
Least-squares matrix: full	Hydrogen site location: inferred from neighbouring sites
*R*[*F*^2^ > 2σ(*F*^2^)] = 0.038	H-atom parameters constrained
*wR*(*F*^2^) = 0.106	*w* = 1/[σ^2^(*F*_o_^2^) + (0.0324*P*)^2^ + 0.5658*P*] where *P* = (*F*_o_^2^ + 2*F*_c_^2^)/3
*S* = 1.01	(Δ/σ)_max_ < 0.001
3438 reflections	Δρ_max_ = 0.15 e Å^−^^3^
254 parameters	Δρ_min_ = −0.24 e Å^−^^3^
0 restraints	Extinction correction: *SHELXL97* (Sheldrick, 2008), Fc^*^=kFc[1+0.001xFc^2^λ^3^/sin(2θ)]^-1/4^
Primary atom site location: structure-invariant direct methods	Extinction coefficient: 0.0036 (6)

### Special details

**Table:**

Geometry. All e.s.d.'s (except the e.s.d. in the dihedral angle between two l.s. planes) are estimated using the full covariance matrix. The cell e.s.d.'s are taken into account individually in the estimation of e.s.d.'s in distances, angles and torsion angles; correlations between e.s.d.'s in cell parameters are only used when they are defined by crystal symmetry. An approximate (isotropic) treatment of cell e.s.d.'s is used for estimating e.s.d.'s involving l.s. planes.
Refinement. Refinement of *F*^2^ against ALL reflections. The weighted *R*-factor *wR* and goodness of fit *S* are based on *F*^2^, conventional *R*-factors *R* are based on *F*, with *F* set to zero for negative *F*^2^. The threshold expression of *F*^2^ > σ(*F*^2^) is used only for calculating *R*-factors(gt) *etc*. and is not relevant to the choice of reflections for refinement. *R*-factors based on *F*^2^ are statistically about twice as large as those based on *F*, and *R*- factors based on ALL data will be even larger.

### Fractional atomic coordinates and isotropic or equivalent isotropic displacement parameters (Å^2^)

**Table:**

	*x*	*y*	*z*	*U*_iso_*/*U*_eq_	
Cl1	0.28670 (5)	0.3365 (2)	0.13385 (4)	0.0869 (4)	
O1	0.42313 (10)	1.2588 (4)	0.50098 (10)	0.0631 (6)	
O2	0.37700 (9)	0.9028 (4)	0.43966 (8)	0.0556 (6)	
O3	0.06269 (9)	0.9926 (4)	0.41172 (8)	0.0526 (5)	
O4	0.05353 (10)	1.1201 (5)	0.30620 (8)	0.0639 (6)	
N1	0.51710 (12)	1.3321 (5)	0.40646 (11)	0.0548 (6)	
H1	0.5250	1.4524	0.4375	0.066*	
N2	0.23923 (11)	0.7752 (5)	0.39523 (11)	0.0527 (7)	
N3	−0.05067 (11)	1.4950 (5)	0.33134 (10)	0.0529 (7)	
H3	−0.0412	1.5110	0.2917	0.063*	
C1	0.55231 (16)	1.3079 (7)	0.35432 (15)	0.0637 (9)	
H1A	0.5892	1.4169	0.3462	0.076*	
C2	0.52445 (16)	1.0957 (7)	0.31537 (15)	0.0642 (9)	
H2	0.5388	1.0351	0.2761	0.077*	
C3	0.47069 (15)	0.9876 (7)	0.34556 (14)	0.0561 (8)	
H3A	0.4424	0.8414	0.3301	0.067*	
C4	0.46687 (14)	1.1355 (6)	0.40230 (13)	0.0464 (7)	
C5	0.42220 (14)	1.1110 (7)	0.45249 (14)	0.0472 (7)	
C6	0.32809 (13)	0.8638 (7)	0.48606 (12)	0.0538 (8)	
H6A	0.3082	1.0432	0.4961	0.065*	
H6B	0.3494	0.7831	0.5262	0.065*	
C7	0.27484 (14)	0.6671 (6)	0.45514 (13)	0.0541 (8)	
H7A	0.2958	0.4891	0.4458	0.065*	
H7B	0.2425	0.6301	0.4862	0.065*	
C8	0.25169 (13)	0.6761 (6)	0.33371 (13)	0.0469 (7)	
C9	0.30389 (14)	0.4884 (7)	0.32549 (14)	0.0570 (8)	
H9	0.3324	0.4320	0.3616	0.068*	
C10	0.31402 (15)	0.3851 (7)	0.26484 (16)	0.0645 (9)	
H10	0.3481	0.2547	0.2607	0.077*	
C11	0.27436 (16)	0.4726 (7)	0.21056 (14)	0.0575 (8)	
C12	0.22420 (15)	0.6637 (7)	0.21674 (14)	0.0600 (9)	
H12	0.1978	0.7265	0.1798	0.072*	
C13	0.21230 (14)	0.7647 (7)	0.27770 (14)	0.0556 (8)	
H13	0.1776	0.8930	0.2812	0.067*	
C14	0.18334 (13)	0.9640 (6)	0.40279 (13)	0.0529 (8)	
H14A	0.1808	1.1052	0.3686	0.063*	
H14B	0.1910	1.0604	0.4444	0.063*	
C15	0.11705 (13)	0.8046 (6)	0.39957 (14)	0.0530 (8)	
H15A	0.1076	0.7194	0.3568	0.064*	
H15B	0.1204	0.6548	0.4318	0.064*	
C16	0.03441 (14)	1.1389 (6)	0.36010 (13)	0.0461 (7)	
C17	−0.01934 (13)	1.3157 (6)	0.37684 (12)	0.0423 (7)	
C18	−0.04881 (14)	1.3549 (6)	0.43337 (13)	0.0534 (8)	
H18	−0.0375	1.2621	0.4727	0.064*	
C19	−0.09868 (15)	1.5583 (7)	0.42135 (15)	0.0643 (9)	
H19	−0.1270	1.6252	0.4510	0.077*	
C20	−0.09841 (15)	1.6418 (7)	0.35789 (15)	0.0623 (9)	
H20	−0.1265	1.7770	0.3368	0.075*	

### Atomic displacement parameters (Å^2^)

**Table:**

	*U*^11^	*U*^22^	*U*^33^	*U*^12^	*U*^13^	*U*^23^
Cl1	0.0873 (7)	0.1160 (8)	0.0615 (5)	0.0033 (6)	0.0274 (5)	−0.0115 (5)
O1	0.0676 (14)	0.0668 (15)	0.0543 (12)	−0.0081 (12)	0.0035 (10)	−0.0143 (12)
O2	0.0497 (12)	0.0689 (15)	0.0488 (12)	−0.0128 (12)	0.0084 (9)	−0.0084 (11)
O3	0.0464 (12)	0.0692 (14)	0.0436 (11)	0.0102 (11)	0.0116 (9)	0.0052 (11)
O4	0.0646 (13)	0.0917 (17)	0.0371 (11)	0.0102 (13)	0.0130 (10)	−0.0033 (11)
N1	0.0536 (15)	0.0539 (16)	0.0562 (15)	−0.0041 (14)	0.0022 (12)	−0.0013 (13)
N2	0.0418 (14)	0.0638 (18)	0.0517 (15)	0.0093 (13)	0.0004 (12)	0.0011 (13)
N3	0.0504 (15)	0.0690 (18)	0.0393 (13)	−0.0003 (14)	0.0051 (12)	0.0025 (13)
C1	0.057 (2)	0.070 (2)	0.065 (2)	−0.0030 (19)	0.0110 (17)	0.011 (2)
C2	0.066 (2)	0.073 (3)	0.0548 (19)	0.000 (2)	0.0137 (17)	−0.0007 (19)
C3	0.056 (2)	0.060 (2)	0.0521 (18)	−0.0013 (18)	0.0028 (15)	0.0004 (17)
C4	0.0407 (17)	0.048 (2)	0.0491 (18)	−0.0010 (16)	−0.0021 (14)	0.0025 (16)
C5	0.0410 (18)	0.050 (2)	0.0480 (18)	0.0039 (16)	−0.0082 (14)	0.0029 (16)
C6	0.0490 (18)	0.069 (2)	0.0436 (17)	−0.0018 (17)	0.0064 (14)	0.0031 (16)
C7	0.0493 (18)	0.060 (2)	0.0545 (18)	−0.0017 (17)	0.0110 (15)	0.0118 (16)
C8	0.0389 (17)	0.051 (2)	0.0515 (18)	−0.0034 (16)	0.0086 (14)	0.0023 (16)
C9	0.0475 (19)	0.068 (2)	0.0551 (19)	0.0104 (17)	0.0031 (15)	0.0016 (17)
C10	0.054 (2)	0.069 (2)	0.073 (2)	0.0122 (19)	0.0160 (17)	0.000 (2)
C11	0.054 (2)	0.068 (2)	0.0529 (19)	−0.0033 (19)	0.0168 (16)	0.0008 (18)
C12	0.056 (2)	0.072 (2)	0.0517 (19)	0.0008 (19)	0.0040 (15)	0.0119 (18)
C13	0.0483 (18)	0.062 (2)	0.0566 (19)	0.0061 (16)	0.0055 (15)	0.0055 (17)
C14	0.0424 (18)	0.059 (2)	0.0574 (18)	0.0011 (17)	0.0052 (14)	−0.0062 (16)
C15	0.0446 (18)	0.056 (2)	0.0594 (18)	0.0049 (17)	0.0116 (14)	0.0029 (16)
C16	0.0433 (17)	0.057 (2)	0.0384 (17)	−0.0065 (16)	0.0047 (14)	−0.0002 (16)
C17	0.0409 (16)	0.0530 (19)	0.0324 (14)	0.0004 (15)	0.0010 (12)	0.0011 (14)
C18	0.0558 (19)	0.065 (2)	0.0404 (16)	0.0045 (18)	0.0113 (14)	0.0016 (16)
C19	0.062 (2)	0.075 (3)	0.058 (2)	0.013 (2)	0.0159 (17)	−0.0068 (18)
C20	0.053 (2)	0.065 (2)	0.067 (2)	0.0095 (18)	0.0005 (17)	0.0014 (19)

### Geometric parameters (Å, °)

**Table:**

Cl1—C11	1.748 (3)	C6—H6B	0.9700
O1—C5	1.219 (3)	C7—H7A	0.9700
O2—C5	1.346 (3)	C7—H7B	0.9700
O2—C6	1.447 (3)	C8—C9	1.394 (4)
O3—C16	1.345 (3)	C8—C13	1.395 (4)
O3—C15	1.446 (3)	C9—C10	1.377 (4)
O4—C16	1.214 (3)	C9—H9	0.9300
N1—C1	1.347 (3)	C10—C11	1.368 (4)
N1—C4	1.366 (3)	C10—H10	0.9300
N1—H1	0.8600	C11—C12	1.366 (4)
N2—C8	1.398 (3)	C12—C13	1.387 (4)
N2—C14	1.452 (3)	C12—H12	0.9300
N2—C7	1.455 (3)	C13—H13	0.9300
N3—C20	1.341 (3)	C14—C15	1.520 (4)
N3—C17	1.370 (3)	C14—H14A	0.9700
N3—H3	0.8600	C14—H14B	0.9700
C1—C2	1.370 (4)	C15—H15A	0.9700
C1—H1A	0.9300	C15—H15B	0.9700
C2—C3	1.392 (4)	C16—C17	1.432 (4)
C2—H2	0.9300	C17—C18	1.370 (3)
C3—C4	1.372 (4)	C18—C19	1.391 (4)
C3—H3A	0.9300	C18—H18	0.9300
C4—C5	1.438 (4)	C19—C20	1.367 (4)
C6—C7	1.507 (4)	C19—H19	0.9300
C6—H6A	0.9700	C20—H20	0.9300
			
C5—O2—C6	116.6 (2)	C10—C9—H9	119.4
C16—O3—C15	116.3 (2)	C8—C9—H9	119.4
C1—N1—C4	109.3 (3)	C11—C10—C9	120.6 (3)
C1—N1—H1	125.3	C11—C10—H10	119.7
C4—N1—H1	125.3	C9—C10—H10	119.7
C8—N2—C14	120.9 (2)	C12—C11—C10	119.6 (3)
C8—N2—C7	122.3 (2)	C12—C11—Cl1	120.1 (2)
C14—N2—C7	116.2 (2)	C10—C11—Cl1	120.3 (3)
C20—N3—C17	109.7 (2)	C11—C12—C13	120.5 (3)
C20—N3—H3	125.1	C11—C12—H12	119.7
C17—N3—H3	125.1	C13—C12—H12	119.7
N1—C1—C2	108.4 (3)	C12—C13—C8	120.8 (3)
N1—C1—H1A	125.8	C12—C13—H13	119.6
C2—C1—H1A	125.8	C8—C13—H13	119.6
C1—C2—C3	107.1 (3)	N2—C14—C15	111.4 (2)
C1—C2—H2	126.4	N2—C14—H14A	109.4
C3—C2—H2	126.4	C15—C14—H14A	109.4
C4—C3—C2	107.7 (3)	N2—C14—H14B	109.4
C4—C3—H3A	126.1	C15—C14—H14B	109.4
C2—C3—H3A	126.1	H14A—C14—H14B	108.0
N1—C4—C3	107.4 (3)	O3—C15—C14	110.6 (2)
N1—C4—C5	121.0 (3)	O3—C15—H15A	109.5
C3—C4—C5	131.6 (3)	C14—C15—H15A	109.5
O1—C5—O2	122.5 (3)	O3—C15—H15B	109.5
O1—C5—C4	125.7 (3)	C14—C15—H15B	109.5
O2—C5—C4	111.8 (3)	H15A—C15—H15B	108.1
O2—C6—C7	107.1 (2)	O4—C16—O3	122.7 (3)
O2—C6—H6A	110.3	O4—C16—C17	125.2 (3)
C7—C6—H6A	110.3	O3—C16—C17	112.1 (2)
O2—C6—H6B	110.3	N3—C17—C18	107.0 (3)
C7—C6—H6B	110.3	N3—C17—C16	120.0 (2)
H6A—C6—H6B	108.5	C18—C17—C16	133.0 (3)
N2—C7—C6	113.8 (2)	C17—C18—C19	107.7 (3)
N2—C7—H7A	108.8	C17—C18—H18	126.1
C6—C7—H7A	108.8	C19—C18—H18	126.1
N2—C7—H7B	108.8	C20—C19—C18	107.4 (3)
C6—C7—H7B	108.8	C20—C19—H19	126.3
H7A—C7—H7B	107.7	C18—C19—H19	126.3
C9—C8—C13	117.3 (3)	N3—C20—C19	108.2 (3)
C9—C8—N2	121.9 (3)	N3—C20—H20	125.9
C13—C8—N2	120.8 (3)	C19—C20—H20	125.9
C10—C9—C8	121.2 (3)		
			
C4—N1—C1—C2	0.6 (3)	C9—C10—C11—C12	−0.2 (5)
N1—C1—C2—C3	−0.3 (3)	C9—C10—C11—Cl1	−179.0 (2)
C1—C2—C3—C4	−0.1 (3)	C10—C11—C12—C13	−1.4 (5)
C1—N1—C4—C3	−0.7 (3)	Cl1—C11—C12—C13	177.5 (2)
C1—N1—C4—C5	178.8 (3)	C11—C12—C13—C8	0.7 (5)
C2—C3—C4—N1	0.5 (3)	C9—C8—C13—C12	1.4 (4)
C2—C3—C4—C5	−178.9 (3)	N2—C8—C13—C12	−179.2 (3)
C6—O2—C5—O1	0.9 (4)	C8—N2—C14—C15	78.5 (3)
C6—O2—C5—C4	−178.3 (2)	C7—N2—C14—C15	−93.4 (3)
N1—C4—C5—O1	1.7 (4)	C16—O3—C15—C14	84.3 (3)
C3—C4—C5—O1	−179.0 (3)	N2—C14—C15—O3	176.0 (2)
N1—C4—C5—O2	−179.1 (2)	C15—O3—C16—O4	−1.2 (4)
C3—C4—C5—O2	0.2 (4)	C15—O3—C16—C17	179.2 (2)
C5—O2—C6—C7	168.1 (2)	C20—N3—C17—C18	−0.2 (3)
C8—N2—C7—C6	104.4 (3)	C20—N3—C17—C16	−179.1 (3)
C14—N2—C7—C6	−83.8 (3)	O4—C16—C17—N3	−4.3 (4)
O2—C6—C7—N2	−61.2 (3)	O3—C16—C17—N3	175.3 (2)
C14—N2—C8—C9	−178.2 (3)	O4—C16—C17—C18	177.1 (3)
C7—N2—C8—C9	−6.8 (4)	O3—C16—C17—C18	−3.3 (4)
C14—N2—C8—C13	2.5 (4)	N3—C17—C18—C19	0.5 (3)
C7—N2—C8—C13	173.8 (3)	C16—C17—C18—C19	179.2 (3)
C13—C8—C9—C10	−3.0 (4)	C17—C18—C19—C20	−0.6 (4)
N2—C8—C9—C10	177.7 (3)	C17—N3—C20—C19	−0.1 (3)
C8—C9—C10—C11	2.4 (5)	C18—C19—C20—N3	0.4 (4)

### Hydrogen-bond geometry (Å, °)

**Table:**

*D*—H···*A*	*D*—H	H···*A*	*D*···*A*	*D*—H···*A*
N1—H1···O1^i^	0.86	2.07	2.893 (3)	160
N3—H3···O4^ii^	0.86	2.07	2.891 (3)	158

Symmetry codes: (i) −*x*+1, −*y*+3, −*z*+1; (ii) −*x*, *y*+1/2, −*z*+1/2.
